# Avoiding False-Positive SARS-CoV-2 Rapid Antigen Test Results with Point-of-Care Molecular Testing on Residual Test Buffer

**DOI:** 10.1128/spectrum.00639-22

**Published:** 2022-07-13

**Authors:** Jason J. LeBlanc, Gregory R. McCracken, Barbara Goodall, Todd F. Hatchette, Lisa Barrett, John Ross, Ross J. Davidson, Glenn Patriquin

**Affiliations:** a Department of Pathology and Laboratory Medicine, Division of Microbiology, Nova Scotia Health, Halifax, Nova Scotia, Canada; b Department of Pathology, Dalhousie University, Halifax, Nova Scotia, Canada; c Department of Medicine, Dalhousie University, Halifax, Nova Scotia, Canada; d Department of Microbiology and Immunology, Dalhousie University, Halifax, Nova Scotia, Canada; e Praxes Medical Group, Halifax, Nova Scotia, Canada; Quest Diagnostics Nichols Institute

**Keywords:** COVID-19, SARS-CoV-2, rapid, antigen, buffer, false-positive, PCR, POC, residual, specificity, IDNOW, Panbio, rapid tests, sensitivity

## Abstract

Antigen-based rapid diagnostic tests (Ag-RDTs) have been widely used for the detection of SARS-CoV-2 during the coronavirus disease 2019 (COVID-19) pandemic. In settings of low disease prevalence, such as asymptomatic community testing, national guidelines recommend confirmation of positive Ag-RDT results with a nucleic acid amplification test (NAAT). This often requires patients to be recalled for repeat specimen recollection and subsequent testing in reference laboratories. This project assessed the use of a point-of-care molecular NAAT for SARS-CoV-2 detection (i.e., ID NOW), which was performed on-site at a volunteer-led asymptomatic community testing site on the residual test buffer (RTB) from positive Ag-RDTs. The ID NOW NAAT assay was performed on RTB from two Ag-RDTs: the Abbott Panbio and BTNX Rapid Response assays. Results of ID NOW were compared to real-time RT-PCR at a reference laboratory. Along with investigations into the clinical performance of ID NOW on RTB, analytical specificity was assessed with a panel of various respiratory organisms. Of the Ag-RDTs results evaluated, all 354 Ag-RDTs results characterized as true positives by RT-PCR were accurately identified with ID NOW testing of RTB. No SARS-CoV-2 detections by ID NOW were observed from 10 specimens characterized as false-positive Ag-RDTs, or from contrived specimens with various respiratory organisms. The use of on-site molecular testing on RTB provides a suitable option for rapid confirmatory testing of positive Ag-RDTs, thereby obviating the need for specimen recollection for molecular testing at local reference laboratories.

**IMPORTANCE** During the COVID-19 pandemic, rapid antigen tests have been widely used for the detection of SARS-CoV-2. These simple devices allow rapid test results. However, false-positive results may occur. As such, individuals with positive rapid tests often must return to testing centers to have a second swab collected, which is then transported to a specialized laboratory for confirmation using molecular tests. As an alternative to requiring a repeat visit and a prolonged turn-around time for result confirmation, this project evaluated whether the leftover material from rapid antigen tests could be confirmed directly on a portable point-of-care molecular instrument. Using this approach, molecular confirmation of positive antigen tests could be performed in less than 15 min, and the results were equivalent to laboratory-based confirmation. This procedure eliminates the need for individuals to return to testing centers following a positive rapid antigen test and ensures accurate antigen test results through on-site confirmation.

## INTRODUCTION

With their simplicity, speed, and scalability, antigen-based rapid diagnostic tests (Ag-RDTs) have been deployed worldwide to facilitate SARS-CoV-2 detection (1–4). Ag-RDT positive results have been associated with the ability to culture SARS-CoV-2 *in vitro* or viral loads consistent with a transmissible virus. Therefore, Ag-RDTs have been used as a surrogate for SARS-CoV-2 communicability (5–9). Nova Scotia was the first Canadian province to implement Ag-RDTs for self-perceived asymptomatic individuals in low-barrier volunteer-led community testing centers, to identify individuals at high risk of transmitting SARS-CoV-2 that might otherwise have gone unnoticed (10, 11). Following national guidelines, individuals with positive Ag-RDTs were asked to return to testing centers for specimen recollection, and confirmatory testing using nucleic acid amplification tests (NAATs) performed at local reference laboratories. To streamline confirmation of positive Ag-RDTs, direct NAAT testing on the Ag-RDT residual test buffer (RTB) (10, 12) was evaluated.

Like others (12), our previous study demonstrated high sensitivity for SARS-CoV-2 detection using RTB from nasopharyngeal and nasal swab collections (10). RTB obviated the need for specimen recollection for NAAT-based confirmation of Ag-RDT results, but RTB processing remained at the reference laboratory. To further optimize community testing strategies, this project evaluated a portable NAAT-based rapid diagnostic test (NAAT-RDT) for on-site confirmation of Ag-RDTs-positive results at the community testing centers (13–17). The COVID-19 assay on the Abbott ID NOW instrument is a NAAT-RDT that uses isothermal technology that is amenable to point-of-care applications (4). This NAAT-RDT is simple and provides rapid results with sensitivity and specificity comparable to other NAATs, but its single-specimen processing limits its scalability for testing large populations (13–17). Instead, this NAAT-RDT was evaluated for rapid confirmation of SARS-CoV-2 using RTB (10, 12) from positive Ag-RDTs, at the site of sample collection (Table 1).

**TABLE 1:** Summary of ID NOW results from all study phases

Category^*a*^			Positive ID NOW results^*b*^			
			Nasal (*n* = 164)	Throat (*n* = 93)	Combined nasal/throat (*n* = 162)	Total (*n* = 419)
Antigen status	True positive (Ag+/PCR+)		100.0% (132/132)	100.0% (66/66)	100% (156/156)	100.0% (354/354)
	False positive (Ag+/PCR−)		0.0% (0/4)	NA	0.0% (0/6)	0.0% (0/10)
	False negative (Ag−/PCR+)		82.1% (23/28)	92.6% (25/27)	NA	87.3% (48/55)
Antigen score	Ag+/PCR+	3+	100.0% (32/32)	100.0% (13/13)	100.0% (41/41)	100.0% (86/86)
		2+	100.0% (43/43)	100.0% (22/22)	100.0% (52/52)	100.0% (117/117)
		1+	100.0% (36/36)	100.0% (19/19)	100.0% (33/33)	100.0% (88/88)
		+/−	100.0% (21/21)	100.0% (12/12)	100.0% (30/30)	100.0% (63/63)
	Ag+/PCR−	1+	0.0% (0/1)	NA	0.0% (0/2)	0.0% (0/3)
		+/−	0.0% (0/3)	NA	0.0% (0/4)	0.0% (0/7)
Ct value^*c*^	Ag+/PCR+	<25	100.0% (28/28)	100.0% (6/6)	100.0% (62/62)	100.0% (96/96)
		25 to <30	100.0% (58/58)	100.0% (30/30)	100.0% (68/68)	100.0% (156/156)
		≥30	100.0% (46/46)	100.0% (30/30)	100.0% (26/26)	100.0% (102/102)
	Ag−/PCR+	25 to <30	NA	100.0% (4/4)	NA	100.0% (4/4)
		≥30	82.1% (23/28)	91.3% (21/23)	NA	86.3% (44/51)

a Categories represent a stratification of specimens with Ag-RDT positive (Ag+) or Ag-RDT negative (Ag−) results, along with the results of the reference NAAT (RT-PCR using the Taqpath assay, denoted as either positive [PCR+] or negative [PCR−]).

b ID Now results for individual tests and project phases are provided in Table S1 to S3.

c Ct values were categorized based on the N gene of the TaqPath real-time RT-PCR. Abbreviations: antigen (Ag); antigen-based rapid diagnostic test (Ag-RDT); threshold cycle (Ct); nucleic acid amplification test (NAAT); residual test buffer (RTB).

## RESULTS

Of the Ag-RDTs results evaluated, all 354 positive results characterized as true positives by RT-PCR were accurately identified with ID NOW testing of RTB, and the 10 false-positive Ag-RDTs results were correctly identified as negative (Table 1). Because the performance of a test can vary with many parameters (3), the data were divided into different categories. Briefly, the ID NOW was positive for all Ag-RDT positive RTB samples, regardless of anatomical site of collection, Ag-RDT method used, antigen score, or RT-PCR threshold cycle (Ct) value (Table S1 to S3). Compared to the RT-PCR reference method, no false-positives were identified with ID NOW, suggesting high specificity. These data support the use of the NAAT-RDT to quickly rule in SARS-CoV-2 using RTB from positive Ag-RDTs, thereby ruling out false-positive Ag-RDT reactions. However, further testing was needed to verify if false-positive Ag-RDTs would be negative with the ID NOW assay, given none were observed during the Investigation of Sensitivity of Nose and Throat (ISNOT) period of the project.

To further assess specificity, two strategies were undertaken. First, highly concentrated nucleic acids from various respiratory microorganisms were spiked into 300 μL Panbio buffer and tested with the ID NOW assay. The assay detected a variety of SARS-CoV-2 lineages, but no cross-reactions were observed with other respiratory organisms (Table S4). For 2 weeks following the ISNOT project, RTB from positive Ag-RDT reactions was then subjected to ID NOW and RT-PCR testing (Table S3). Of 3676 individuals tested by Ag-RDTs, 147 were positive, and 137 of these were positive by both ID NOW and RT-PCR. The 10 false-positive Ag-RDTs compared to RT-PCR were also negative by ID NOW (Table 1 and Table S3). Consistent with our previous study (10), false-positive Ag-RDTs were described as having barely visible target bands, with antigen scores +/− or 1+ (Table 1 and Table S3).

## DISCUSSION

**FIG 1:**
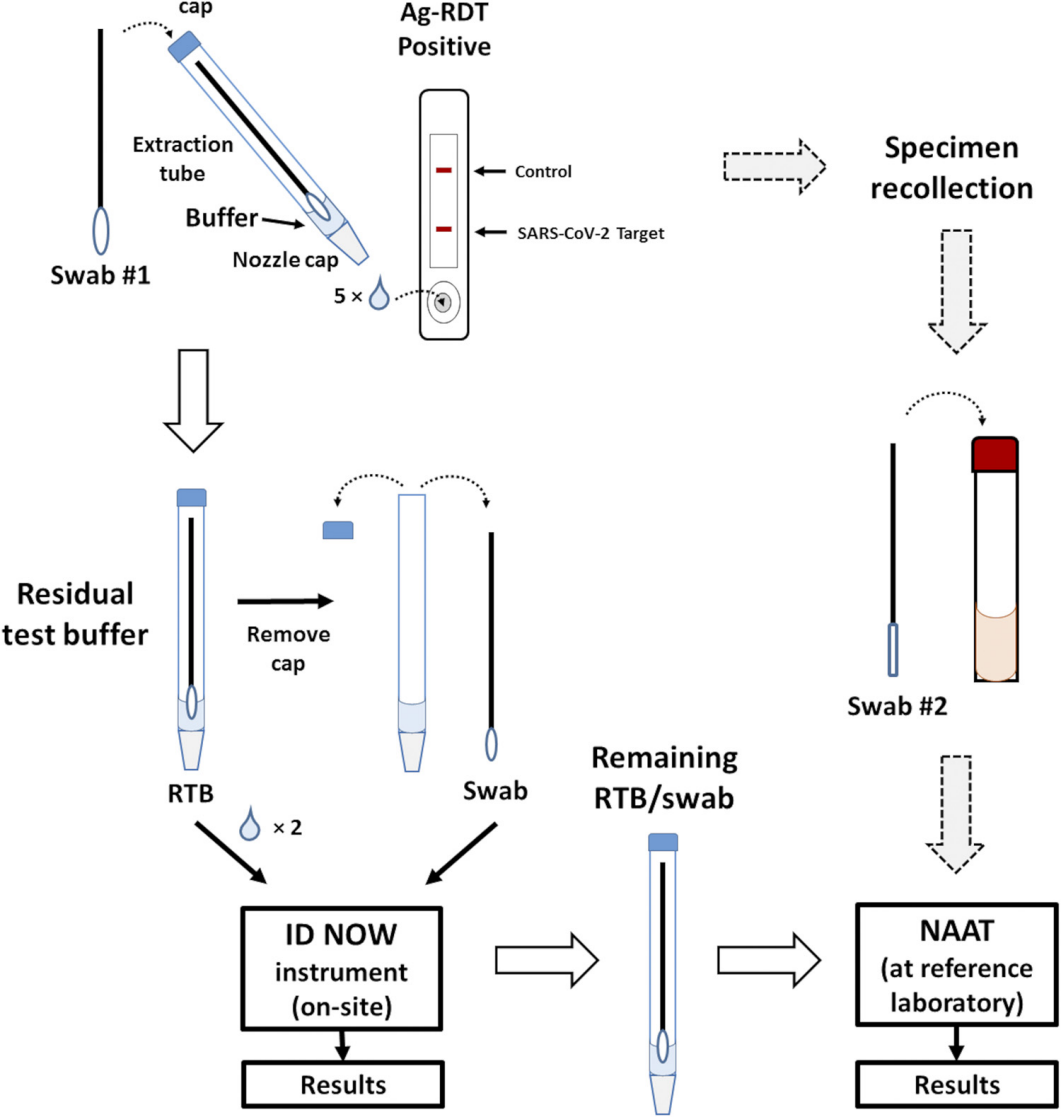
Specimen flow for the evaluation. Following collection, the swab was placed into an extraction tube prefilled with 11 to 12 drops of the buffer. The tube was pinched to help extract the respiratory secretions from the swab, which in turn is rotated into the buffer. The tube was then capped on top, and the bottom nozzle cap was removed. Five drops were placed into the sample well of the lateral flow device, and after 15 to 20 min, the results were read. When a positive Ag-RDT was obtained (i.e., the presence of both the control and target bands), national guidelines recommended specimen collection and submission to reference laboratories for confirmation (see large gray dashed arrows). In a previous study (10), RT-PCR on residual test buffer (RTB) was validated against specimens recollected following positive Ag-RDT results and demonstrated high accuracy. In this study, RTB tested at the site of collection on an ID NOW instrument was validated against RTB tested by RT-PCR at a reference laboratory. As depicted in an illustration of the Panbio COVID-19 Ag Rapid Test Device, the process would be nearly identical for the BTNX Rapid Response kit with few exceptions, including the number of drops and devices used for testing.

The ISNOT project (1) validated the use of nose/throat collections for Ag-RDT, and given participants were being enrolled for method evaluation, the performance of a point-of-care NAAT-RDT (i.e., ID NOW) was compared against RT-PCR performed at a reference laboratory using the residual buffer from Ag-RDT testing. Our previous study had validated the use of RTB with real-time RT-PCR in a clinical laboratory for nasopharyngeal and nasal swab collections (100.0% and 98.7% sensitivity, respectively), but it was hypothesized that testing RTB on a portable NAAT instrument (i.e., ID NOW) could provide further benefits by allowing rapid method for Ag-RDT confirmation at the site of collection, thereby obviating the need specimen recollection and testing delays that occur from offsite molecular confirmation of Ag-RDT results at reference laboratories (Fig. 1).

Specificity was the focus of the investigation RTB with the ID NOW, given that false-positive reactions can occur following Ag-RDT positive results, particularly in a setting of low disease prevalence such as community testing. Overall, ID NOW confirmed 354 true positive Ag-RDT results, ruled out 10 false-positives, and there was no cross-reactivity with other respiratory organisms. The proportion of false-positives observed (10/5148 or 0.2%) is consistent with manufacturer and literature claims, where false-positive reactions are rare at approximately 0.4% (10, 18, 19).

Sensitivity analyses would require IDNOW and RT-PCR testing on all Ag-RDT RTB negative specimens, which would not typically be performed in community-based surveillance. Some sensitivity data were captured during the ISNOT quality initiative (11), as the ID NOW was performed in parallel on Ag-RDT RTB from paired swabs samples from positive individuals. As such, some specimens were negative by Ag-RDT for one swab type of the paired collection, but positive results were obtained by ID NOW and/or RT-PCR. While Ag-RDTs appear less sensitive than NAATs (as seen in Table S1 and S2), it has been argued that the additional detections by NAATs often represent remnant RNA from resolved infections, when the risk for transmission is low (20–22). Alternatively, it may represent periods of early infection that are short-lived in population-based testing and can be mitigated by frequent testing over time with Ag-RDTs (20–22). Importantly, ID NOW confirmed all true positive Ag-RDTs, as well as detected 88.2% (30/34) and 85.7% (18/21) of negative Panbio and BTNX Ag-RDT RTBs that tested positive by RT-PCR, respectively (Table S1 and S2). Discrepant results between ID NOW and RT-PCR were in specimens with Ct values ≥30 (suggesting low viral loads). In sum, while the ID NOW may not be as sensitive as some other NAATs (13–17), the ID NOW was found to be sufficiently sensitive to be used as a confirmatory method for Ag-RDTs, as Ag-RDT themselves would be less sensitive relative to a NAAT comparator (3).

The applications of rapid NAAT-RDT confirmation of positive Ag-RDTs on-site using RTB obviates the need for individuals to return for repeat specimen collection for NAAT testing at local reference laboratories, as well as the need for trained personnel for shipping biological samples to reference laboratories. With recent surges of SARS-CoV-2 activity with the highly transmissible SARS-CoV-2 Omicron variant, many clinical laboratories were overwhelmed with high testing demands, hampering their ability to support confirmation for Ag-RDT-positive results. Given wide community spread, and the low proportion of false-positive results during this period of high disease prevalence, NAAT-based confirmation of Ag-RDT results was not prioritized. However, in the wake of pandemic waves as disease prevalence decreases, the possibility of false-positive Ag-RDT increases, and confirmatory testing for Ag-RDT will again become important to consider (23, 24). The use of RTB testing with NAAT-RDTs provides a feasible and accurate option for rapid confirmatory testing of positive Ag-RDTs on-site at community testing sites. Since performing this evaluation, and due to its simplicity and benefits afforded, the ID NOW was implemented for confirmatory testing using RTB at community testing sites in Nova Scotia.

## MATERIALS AND METHODS

### Specimen collection and Ag-RDT testing.

The assessment was performed in two stages on asymptomatic individuals presenting to urban rapid testing sites. The first overlap with the Investigation of Sensitivity of Nose and Throat (ISNOT) project was designed to compare SARS-CoV-2 1472 Ag-RDT results from self-administered nasal and throat collections (1) (Table S1 and S2), and the second was an extension of the project where additional positive 3676 Ag-RDTs were tested over a subsequent 2-week period to identify false-positive antigen results (Table S3). In both cases, Ag-RDT self-testing using the Panbio COVID-19 Ag Rapid Test Device (Abbott Rapid Diagnostics, Jena, Germany) or the Rapid Response COVID-19 Antigen Rapid Test Device (BTNX Inc., Markham, ON) was used. Ag-RDTs were interpreted according to the manufacturer’s instructions, and SARS-CoV-2 target bands were graded with scores of 0 (negative), +/− (barely visible), or 1+, 2+, or 3+ relative to the intensity of the control band. RTB from any positive Ag-RDT was subjected to both NAATs.

### NAAT on RTB.

The COVID-19 ID Now assay (Abbott Diagnostics, Scarborough, MA) was performed on-site following manufacturer instructions for swab-based collections, except for two drops of RTB that were added to the sample chamber before processing the original Ag-RDT collection swab (Fig. 1). The remaining RTB and swab were transported to a central laboratory in the Ag-RDT reaction tube, and 200 μL of viral transport medial (VTM) (Rodoxica, Little Rock, AR) was added to the tube (to ensure sufficient volumes for NAAT testing). Following vortexing for 10 s, 200 μL of VTM/RTB fluid was subjected to a total nucleic acid extraction (TNA) on a MagNA Pure 96 or LC 2.0 instrument (Roche Diagnostics ltd., Roltkreuz, Switzerland), and 5 μL of the 50 μL of eluted TNAs were used as the template for real-time RT-PCR using the TaqPath COVID-19 Combo kit (Life Technologies Corp., Frederick, MD).

### Ethical statement.

This project was part of a quality initiative and was therefore exempt from review by the Nova Scotia Health Research Ethics Board (submission number 1027644). Specimens tested were obtained from consenting participants, and all data related were provided anonymized, deidentified, and used solely with the intent to evaluate the performance characteristics of testing programs used in Nova Scotia.
