# Supplementary material for: Avoiding False-Positive SARS-CoV-2 Rapid Antigen Test Results with Point-of-Care Molecular Testing on Residual Test Buffer
Source: Microbiol Spectr. 2022 Jul 13;10(4):e00639-22. doi: 10.1128/spectrum.00639-22 (PMC9430663; doi:10.1128/spectrum.00639-22)
Supplement: Supplemental file 1 — Supplemental material. Download spectrum.00639-22-s0001.pdf, PDF file, 0.7 MB [file spectrum.00639-22-s0001.pdf]

Table S1. ID NOW results from the RTB of the Panbio Ag-RDT during the ISNOT project.

Category*			Positive ID NOW results			
			Nasal (n=96)	Throat (n=55)	Combined nasal/throat (n=42)	Total (n=193)
Antigen status	True positive (Ag+/PCR+)		100.0% (77/77)	100.0% (40/40)	100.0% (42/42)	100.0% (159/159)
	False positive (Ag+/PCR-)		N/A	N/A	N/A	N/A
	False negative (Ag-/PCR+)		78.9% (15/19)	100.0% (15/15)	N/A	88.2% (30/34)
Antigen score	Ag+/PCR+	3+	100.0% (26/26)	100.0% (13/13)	100.0% (13/13)	100.0% (52/52)
		2+	100.0% (23/23)	100.0% (10/10)	100.0% (16/16)	100.0% (49/49)
		1+	100.0% (20/20)	100.0% (11/11)	100.0% (8/8)	100.0% (39/39)
		+/-	100.0% (8/8)	100.0% (6/6)	100.0% (5/5)	100.0% (19/19)
Ct value**	Ag+/PCR+	<25	100.0% (16/16)	100.0% (2/2)	100.0% (8/8)	100.0% (26/26)
		25 to <30	100.0% (31/31)	100.0% (18/18)	100.0% (22/22)	100.0% (71/71)
		≥30	100.0% (30/30)	100.0% (20/20)	100.0% (12/12)	100.0% (62/62)
	Ag-/PCR+	<25	N/A	N/A	N/A	N/A
		25 to <30	N/A	N/A	N/A	N/A
		≥30	78.9% (15/19)	100.0% (15/15)	N/A	88.2% (30/34)

*Categories represent a stratification of specimens with Ag-RDT positive (Ag+) or Ag-RDT negative (Ag-) results, along with the results of the reference NAAT [RT-PCR using the Taqpath assay - which was either positive (PCR+) or negative (PCR-)]. **Ct values were categorized based on the N gene of the TaqPath real-time RT-PCR. Abbreviations: antigen (Ag); antigen-based rapid diagnostic test (Ag-RDT); threshold cycle (Ct); nucleic acid amplification test (NAAT); residual test buffer (RTB).

Table S2. ID NOW results from the RTB of the BTNX Ag-RDT during the ISNOT project.

Category*			Positive IDNOW results		
			Nasal (n=41)	Throat (n=38)	Total (n=79)
Antigen status	True positive (Ag+/PCR+)		100.0% (32/32)	100.0% (26/26)	100.0% (58/58)
	False positive (Ag+/PCR-)		N/A	N/A	N/A
	False negative (Ag-/PCR+)		88.9% (8/9)	83.3% (10/12)	85.7% (18/21)
Antigen score	Ag+/PCR+	2+	100.0% (16/16)	100.0%(12/12)	100.0% (28/28)
		1+	100.0% (12/12)	100.0% (8/8)	100.0% (20/20)
		+/-	100.0% (4/4)	100.0% (6/6)	100.0% (10/10)
Ct value**	Ag+/PCR+	<25	100.0% (1/1)	100.0% (4/4)	100.0% (5/5)
		25 to <30	100.0% (20/20)	100.0% (12/12)	100.0% (32/32)
		≥30	100.0% (11/11)	100.0% (10/10)	100.0% (21/21)
	Ag-/PCR+	<25	N/A	N/A	N/A
		25 to <30	N/A	100.0% (4/4)***	100.0% (4/4)***
		≥30	88.9% (8/9)	75.0% (6/8)	82.4% (14/17)

*Categories represent a stratification of specimens with Ag-RDT positive (Ag+) or Ag-RDT negative (Ag-) results, along with the results of the reference NAAT [RT-PCR using the Taqpath assay, denoted by either positive (PCR+) or negative (PCR-)]. **Ct values were categorized based on the N gene of the TaqPath real-time RT-PCR. ***Ct values falling into this category were 27.43, 27.71, 29.63, and 29.87. Abbreviations: antigen (Ag); antigen-based rapid diagnostic test (Ag-RDT); threshold cycle (Ct); nucleic acid amplification test (NAAT); residual test buffer (RTB).

Table S3. Summary of the supplemental testing to identify false positive with the Panbio Ag-RDT.

Category*			Positive ID NOW results		
			Nasal (n=27)	Combined nasal/throat (n=120)	Total (n=147)
**Antigen status**	**Ag+/NAAT+**		100.0% (23/23)	100.0% (114/114)	100.0% (137/137)
	**Ag+/NAAT-**		0.0% (0/4)	0.0% (0/6)	0.0% (0/10)
	**Ag-/NAAT+**		N/A	N/A	N/A
**Antigen score**	**Ag+/NAAT+**	**3+**	100.0% (6/6)	100.0% (28/28)	100.0% (34/34)
		**2+**	100.0% (4/4)	100.0% (36/36)	100.0% (40/40)
		**1+**	100.0% (4/4)	100.0% (25/25)	100.0% (29/29)
		**+/-**	100.0% (9/9)	100.0% (25/25)	100.0% (34/34)
	**Ag+/NAAT-**	**3+**	N/A	N/A	N/A
		**2+**	N/A	N/A	N/A
		**1+**	0.0% (0/1)	0.0% (0/2)	0.0% (0/3)
		**+/-**	0.0% (0/3)	0.0% (0/4)	0.0% (0/7)
**Ct value****	**Ag+/NAAT+**	**<25**	100.0% (11/11)	100.0% (54/54)	100.0% (65/65)
		**25 to <30**	100.0% (7/7)	100.0% (46/46)	100.0% (53/53)
		**≥30**	100.0% (5/5)	100.0% (14/14)	100.0% (19/19)

*Categories represent a stratification of specimens with Ag-RDT positive (Ag+) or Ag-RDT negative (Ag-) results, along with the results of the reference NAAT (RT-PCR using the Taqpath assay) which was either positive (NAAT+) or negative (NAAT-). **Ct values were categorized based on the N gene of the TaqPath real-time RT-PCR. Abbreviations: antigen (Ag); antigen-based rapid diagnostic test (Ag-RDT); threshold cycle (Ct); nucleic acid amplification test (NAAT); residual test buffer (RTB).

7

Table S4. Analytical specificity panel for evaluation of the ID NOW COVID-19 assay.

Microorganism	ID NOW result
**SARS-CoV-2 lineages**	
SARS-CoV-2 (lineage A.1)	POS
SARS-CoV-2 (lineage B.1.438)	POS
SARS-CoV-2 (lineage B.1.1.157)	POS
SARS-CoV-2, alpha (lineage B.1.1.7)	POS
SARS-CoV-2, beta (lineage B.1.351)	POS
SARS-CoV-2, gamma (lineage P.1)	POS
SARS-CoV-2, delta (lineage B.1.617.2)	POS
SARS-CoV-2, epsilon (lineage B.1.427)	POS
SARS-CoV-2, iota (lineage B.1.1.526)	POS
SARS-CoV-2, lambda (lineage C.37)	POS
SARS-CoV-2, omicron (lineage B.1.1.529)	POS
**Other human coronaviruses (hCoV)**	
SARS-CoV-1	NEG
MERS-CoV	NEG
hCoV, 229E	NEG
hCoV, OC43	NEG
hCoV, NL63	NEG
hCoV, HKU1	NEG
**Other respiratory viruses**	
Influenza A virus (FluA), subtype H3N2	NEG
Influenza A virus (FluA), subtype H3N2	NEG
Influenza B virus (FluB), Yamagata lineage	NEG
Influenza B virus (FluB), Victoria lineage	NEG
Respiratory syncytial virus (RSV-A), type A	NEG
Respiratory syncytial virus (RSV-B), type B	NEG
Parainfluenza virus, type 1 (P1V)	NEG
Parainfluenza virus, type 2 (P2V)	NEG
Parainfluenza virus, type 3 (P3V)	NEG
Parainfluenza virus, type 4 (P4V)	NEG
Human metapneumovirus (hMPV)	NEG
Human adenovirus (hAdV), serogroup A	NEG
Human adenovirus (hAdV), serogroup B	NEG
Human adenovirus (hAdV), serogroup C	NEG
Human adenovirus (hAdV), serogroup D	NEG
Human adenovirus (hAdV), serogroup E	NEG
Human adenovirus (hAdV), serogroup F	NEG

Human rhinovirus, A (HRV-A)	NEG
Human rhinovirus, B (HRV-B)	NEG
Human enterovirus (HEV), echo9	NEG
Human enterovirus (HEV), coxsackievirus A	NEG
Human enterovirus (HEV), paraechovirus	NEG
Human enterovirus (HEV), enterovirus D-68	NEG
Human bocavirus (HBoV)	NEG
**Other viruses**	
Measles virus, genotype A	NEG
Measles virus, genotype B3	NEG
Measles virus, genotype D8	NEG
Mumps virus, genotype A	NEG
Mumps virus, genotype C	NEG
Mumps virus, genotype G	NEG
Rubella virus, genotype 1B-F	NEG
Rubella virus, genotype 2B	NEG
Human immunodeficiency virus 1(HIV-1)	NEG
Hepatitis C virus (HCV), genotype 3	NEG
Hepatitis B virus (HBV)	NEG
Herpes simplex virus 1 (HSV-1) [human herpesvirus 1 (HHV-1)]	NEG
Herpes simplex virus 2 (HSV-2) [human herpesvirus 2 (HHV-2)]	NEG
Varicella zoster virus (VZV) [human herpesvirus 3 (HHV-3)]	NEG
Epstein Barr virus (EBV) [human herpesvirus 4 (HHV-4)]	NEG
Cytomegalovirus (CMV) [human herpesvirus 4 (HHV-4)]	NEG
**Other microorganisms**	
Chlamydia pneumoniae	NEG
Haemophilus influenzae	NEG
Legionella pneumophila	NEG
Mycoplasma pneumoniae	NEG
Bordetella pertussis	NEG
Streptococcus pneumoniae	NEG
Streptococcus oralis	NEG
Streptococcus salivarius	NEG
Staphylococcus epidermis	NEG
Staphylococcus aureus	NEG
Pseudomonas aeruginosa	NEG
Mycobacterium tuberculosis	NEG
Pneumocystis jirovecii (PJP)	NEG
Candida albicans	NEG
Corynebacterium sp.	NEG

2
